# Regulatory mechanisms and therapeutic potential of JAB1 in neurological development and disorders

**DOI:** 10.1186/s10020-023-00675-w

**Published:** 2023-06-26

**Authors:** Yu Yang, Ruying Song, Yiming Gao, Hao Yu, Shuai Wang

**Affiliations:** 1 Department of Psychiatry, Jining Medical University, Jianshe South Road No. 45, Jining, Shandong China; 2 Shandong Collaborative Innovation Center for Diagnosis, Treatment and Behavioral Interventions of Mental Disorders, Jining Medical University, Jining, Shandong China

**Keywords:** c-Jun activation domain binding protein-1, COP9 signalosome, Neurodevelopment, Neurological disorders, Regulatory mechanisms

## Abstract

c-Jun activation domain binding protein-1 (JAB1) is a multifunctional regulator that plays vital roles in diverse cellular processes. It regulates AP-1 transcriptional activity and also acts as the fifth component of the COP9 signalosome complex. While JAB1 is considered an oncoprotein that triggers tumor development, recent studies have shown that it also functions in neurological development and disorders. In this review, we summarize the general features of the JAB1 gene and protein, and present recent updates on the regulation of JAB1 expression. Moreover, we also highlight the functional roles and regulatory mechanisms of JAB1 in neurodevelopmental processes such as neuronal differentiation, synaptic morphogenesis, myelination, and hair cell development and in the pathogenesis of some neurological disorders such as Alzheimer’s disease, multiple sclerosis, neuropathic pain, and peripheral nerve injury. Furthermore, current challenges and prospects are discussed, including updates on drug development targeting JAB1.

## Introduction

c-Jun, a component of the activator protein-1 (AP-1) complex, is implicated in a wide range of cellular processes (Herdegen et al. 1997; Shaulian and Karin 2002; Raivich and Behrens 2006). c-Jun activation domain binding protein-1 was identified as a coactivator of c-Jun and hence was originally termed JAB1 (Claret et al. 1996). Two *Arabidopsis* JAB1 homologs, AJH1 and AJH2, were also identified, presenting in both monomeric forms and a constitutive photomorphogenic-9 (COP9) signalosome complex (Kwok et al. 1998). The COP9 signalosomes which were both discovered in plants and animals participate in diverse cellular and developmental processes (Seeger et al. 1998; Wei et al. 1998; Freilich et al. 1999; Qin et al. 2020). Biochemical purification and molecular characterization of the COP9 signalosome from different organisms has identified eight core subunits (Seeger et al. 1998; Mundt et al. 1999; Wei and Deng 1999). JAB1 was identified as the fifth component in the COP9 complex and thereby specified as CSN5 (COP9 signalosome 5) or COPS5 (COP9 signalosome subunit 5) according to different nomenclature (Deng et al. 2000).

JAB1/CSN5 (hereafter JAB1) has been extensively studied in its crucial roles in regulating tumorigenesis. A myriad of evidence demonstrated that JAB1 was upregulated in a variety of malignancies and usually was associated with poor prognosis for human cancers (Sui et al. 2001; Pan et al. 2017; Liu et al. 2019; Wang et al. 2020a). Basically, JAB1 promotes tumor development by propelling cell cycle progression, impairing DNA repair response, regulating cell apoptosis and proliferation, which has been extensively discussed in several reviews (Shackleford and Claret 2010; Pan et al. 2014; Guo et al. 2019; Yuan et al. 2021).

An embryonically lethal phenotype in JAB1-deficient mice suggested that JAB1 was a vital factor in embryogenesis and cell survival. Nullizygous embryos were severely growth-retarded and became inviable before gastrulation (Tomoda et al. 2004). JAB1-deficient embryos exhibited accelerated apoptosis, increased spontaneous DNA damage and homologous recombination (HR) defects which were possibly due to aberrant upregulation of JAB1 targets, such as p27, p53, c-myc, and cyclin E (Tian et al. 2010). JAB1 can also activate c-Jun signaling pathway by potentiating the c-Jun binding specificity to target sites (Claret et al. 1996). c-Jun is spatially differentially expressed in embryonic and adult neural precursor cells (Kawashima et al. 2017) and has been widely recognized as a vital regulator in brain development (Raivich and Behrens 2006; Haeusgen et al. 2009; Raj et al. 2018). Moreover, in recent years, accumulated evidence demonstrated that JAB1 was also functionally implicated in neurodevelopment and the pathologies of some neurological diseases which have been rarely discussed in any review. Herein, we summarize the roles and mechanisms of JAB1 in neurological development and diseases.

## JAB1 gene and protein

**Fig. 1:**
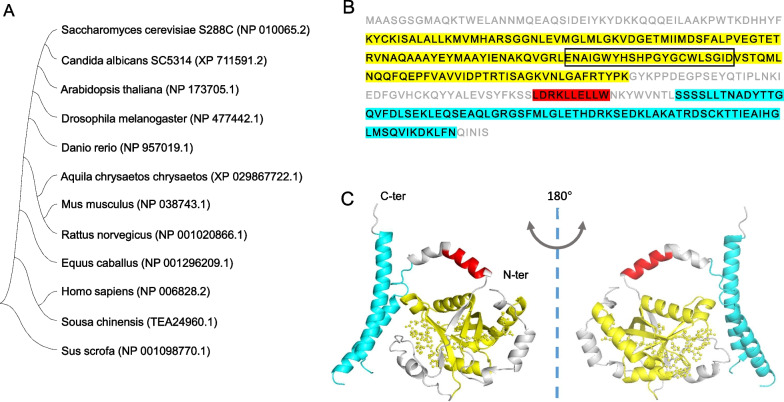
The profile of JAB1 protein. **A** The bootstrap consensus phylogenetic tree of JAB1 proteins from different species. The phylogenetic tree was constructed by using MEGA software version 11.0.13. **B** Human JAB1 amino acid sequences. MPN, NES, and C-terminal domains are shown in yellow, red, and cyan, respectively. JAMM motif is marked by a black box. **C** The structure of human JAB1 is shown based on the data from Protein Data Bank (ID 4D18; https://www.rcsb.org/structure/4D18). MPN, NES, and C-terminal domains are shown in yellow, red, and cyan, respectively. JAMM motif is exhibited by ball and sticks

The human *JAB1* gene, spanning 19,055 bp of genomic DNA, is located on chromosome 8q13.1, which is frequently amplified in some cancers (Fejzo et al. 1998; Rummukainen et al. 2001; Sun et al. 2007). The human *JAB1* gene contains eight exons which assemble into a 1296 bp-length transcript and subsequently encode a protein of 334 amino acids. The JAB1 protein is highly evolutionarily conserved across different species in Eucaryotae (Barth et al. 2016) (Fig. 1A). JAB1 contains a Mpr1-Pad1 N terminal (MPN) domain with a JAB1/MPN/Mov34 metalloenzyme (JAMM) motif (Fig. 1B, C). The MPN domain is associated with isopeptidase/deubiquitinase activities in the ubiquitin-based protein turnover pathways (Schwechheimer and Deng 2001; Tran et al. 2003; Wolf et al. 2003; Duda et al. 2008), and is also supposed to provide a platform for protein interactions (Birol and Echalier 2014). MPN domain also exists in CSN6 while the JAMM (MPN^+^) motif, which functions as a catalytic center of CSN isopeptidase, is only specific in CSN5/JAB1 (Tran et al. 2003; Pan et al. 2022). JAB1 also contains a nuclear export signal (NES) motif ranging from amino acids 233 to 242 (Fig. 1B, C) which resembles the NES sequences in protein kinase I (PKI) and mitogen activated protein kinase (MAPKK). The NES sequence is crucial for the translocation of p27^Kip1^ between nucleus and cytoplasm mediated by chromosomal maintenance 1 (CRM1) (Tomoda et al. 2002). Some reviews have indicated that a specific sequence termed as p27^Kip1^ binding domain (PBD) at the C-terminal of JAB1 was responsible for the interaction between JAB1 and p27^Kip1^ (Shackleford and Claret 2010; Wang et al. 2016a; Yuan et al. 2021). The crystal structure of the human COP9 signalosome revealed a specific C-terminal domain (251-329aa) in JAB1 showing a pronounced effect on CSN integrity (Fig. 1C) (Lingaraju et al. 2014). However, whether the C-terminal domain is implicated in the binding of JAB1 to p27^Kip1^ remains unknown. Moreover, Tomoda et al. reported that N-terminal JAB1(199–334 aa) was highly associated with p27 in glycerol gradient fractionation followed by immunoblotting (Tomoda et al. 2004). Hwang et al. also showed that p27^Kip1^ interacted with the N-terminal region of JAB1 (Hwang et al. 2004). This evidence indicates that the interaction between JAB1 and p27^Kip1^ is probably mediated by multiple sequences and needs to be further elucidated by a structural insight into the JAB1-p27^Kip1^ complex.

## Regulation of JAB1 expression

### Gene amplification and deletion

The expression of JAB1 is known to be regulated at genomic, transcriptional, post transcriptional, translational, and post-translational levels. Gene amplification is a crucial mechanism influencing the expression level of JAB1. The copy number of *JAB1* gene has been shown to be constantly increased in some human cancers and was always correlated with aggressive tumor development and metastatic progression (Fejzo et al. 1998; Rummukainen et al. 2001; Sun et al. 2007). However, the mechanism of *JAB1* amplification remains obscure at present*.* Moreover*,* a clinical report showed that an interstitial deletion of 1.4 Mb-length sequences at the 8q13.1-q13.2 region containing the *JAB1* gene was associated with inferior cerebellar vermian hypoplasia and digital anomalies (Mordaunt et al. 2015). Nevertheless, chromosome deletion-induced JAB1 deficiency rarely happens in physiological or even pathological conditions, which can be partially explained by the vital function of JAB1 in embryonic development (Tomoda et al. 2004).

### Transcription

**Fig. 2:**
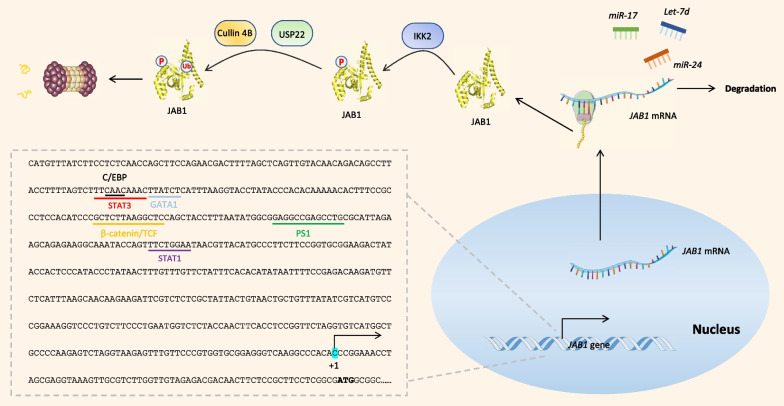
A summary scheme of molecular pathways involved in JAB1 expression. The sequence in the dotted box indicates the promoter of the JAB1 gene. The identified transcription factor binding sites are color-coded. The transcription start site is marked as “+1”. “P” and “Ub” represent the phosphorylation and ubiquitination of target proteins, respectively

Primer extension analysis revealed the transcription start site of the *JAB1* gene at 68 bp upstream of the translation initiation site (ATG) (Fig. 2) (Shackleford et al. 2011). CCAAT/enhancer binding protein (C/EBP), GATA binding protein 1 (GATA1), β-catenin/TCF, Sp1 transcription factor (SP1), signal transducer, and activator of transcription-1 and -3 (Stat1 and Stat3) were demonstrated to regulate *JAB1* transcription by directly binding to their consensus binding sites within the promoter region of the *JAB1* gene (Fig. 2) (Hsu et al. 2007, 2008a, b; Shackleford et al. 2011; Yang et al. 2011; Pan et al. 2017). Moreover, Erb-b2 receptor tyrosine kinase 2 (ERBB2, also termed HER-2/neu) increased β-catenin/TCF-mediated *JAB1* transcription via the AKT signaling pathway (Hsu et al. 2007, 2008a). Troglitazone, a peroxisome proliferator-activated receptor γ (PPARγ) ligand, inhibited *JAB1* promoter activity by suppressing SP1- and TCF4-mediated transcription (Hsu et al. 2008b). The alpha5 nicotinic acetylcholine receptor could also increase *JAB1* transcription by activating STAT3 phosphorylation (Zhu et al. 2022).

### Post-transcription and translation

MicroRNAs (miRNAs) regulate JAB1 expression. For example, miR-24 interacted with both the 3′ untranslated region (UTR) and 5′ UTR of the *JAB1* mRNA, leading to degradation of *JAB1* mRNA and translational suppression (Lal et al. 2009; Wang et al. 2016b). MiR-17 directly targeted *JAB1* mRNA and negatively regulated *JAB1* expression in triple-negative breast cancer cells (Wang et al. 2019b). Let-7d was also reported to directly regulate *JAB1* transcription in breast cancer (Wei et al. 2018).

### Degradation

JAB1 is degraded through the ubiquitin–proteasome pathway. Cullin 4B ubiquitin ligase complex targeted JAB1 for degradation. USP22, a ubiquitin carboxyl-terminal hydrolase, interacted with JAB1 and stabilized JAB1 through deubiquitination (Wang et al. 2020b). Moreover, the degradation of JAB1 was also regulated by protein modifications. Inhibitor kappa B kinase 2 could phosphorylate JAB1 and induced its ubiquitination and degradation (Orel et al. 2010). MAPK activated protein kinase 2 (MK2) phosphorylated JAB1 at Ser177 and facilitated c-Jun recruitment to AP1 binding sites (Chen et al. 2021); however, whether MK2-mediated phosphorylation affects JAB1 degradation remains obscure.

## Molecular functions of JAB1

### Transcriptional co-activation

JAB1 actively takes part in transcriptional co-activation by interacting with transcriptional factors or other transcriptional co-effectors and subsequently influences DNA binding activities of such regulators in a CSN5 independent pathway. JAB1 was first recognized as a transcriptional co-activator because it interacted with c-Jun and regulated AP-1 transcriptional activity (Claret et al. 1996). AP-1 proteins are a cluster of dimeric transcription factors that can be classified into four different subfamilies: Jun (e.g., c-Jun, JunB, JunD), Fos (e.g., c-Fos, Fra1, Fra2, FosB), ATF (e.g., ATF2, ATF3, ATF4 and BATF3) and Maf (e.g., c-Maf, MafA, MafB, MafG) (Yoshitomi et al. 2021). AP-1 proteins are characterized by the presence of a basic leucine zipper (bZip) domain which mediates dimerization of different AP-1 components (Wu et al. 2021). JAB1 interacted with c-Jun and specifically stabilized the c-Jun/JunD-DNA complexes, thereby potentiating c-Jun transactivation (Claret et al. 1996). JAB1-mediated AP-1 activation is also regulated by other JAB1 binding partners. LFA-1 synergized with JAB1 in inducing AP-1 transcriptional activity by regulating redistribution of JAB1 from cytoplasm to nucleus (Bianchi et al. 2000). Hepatopoietin bound to JAB1 and led to potentiation of AP-1 activation (Lu et al. 2002). Similarly, the hepatitis B virus X protein also accelerated AP-1 activation through interaction with JAB1 (Tanaka et al. 2006) (Table 1).

**Table 1:** JAB1-interacting proteins

Functions of Jab1	Target proteins	Effects	References
Transcriptional coactivation	c-Jun	Regulating AP-1 transcriptional activity	Claret et al. (1996)
	Bcl-3	Facilitating the formation of NF-κB/p50 and DNA complex	Dechend et al. (1999)
	HAND2	Augmenting HAND2 transcriptional activity by enhancing HAND2 DNA binding	Dai et al. (2004)
	LFA-1	LFA-1 interacts with the transcriptional co-activator JAB1 to modulate AP-1 activity	Bianchi et al. (2000)
	Brn-2	Regulating Brn-2 DNA-binding activity	Huang et al. (2005)
	5-HT(6)R	5-HT(6)R induced the translocation of Jab1 into the nucleus and increased c-Jun phosphorylation and the interaction between Jab1 and c-Jun	Yun et al. (2010)
	Fank1	Suppressing cell apoptosis by activating the AP-1-induced anti-apoptotic pathway	Wang et al. (2011)
	MIF	Inhibiting AP-1 activity	Kleemann et al. (2000)
	E2F1	Jab1 is an cofactor for E2F1 dependent transcription of apoptotic and mitotic genes	Hallstrom and Nevins (2006), Lu et al. (2011)
DeNEDDylation	Cullins	Modulating the activity of CRLs	Cope et al. (2002), Cope and Deshaies (2003)
	MsrA	Enhancing JAB1’s deneddylase activity	Jiang et al. (2020)
Deubiquitination	ANGPTL2	Inhibiting ANGPTL2 degradation	Xie et al. (2021)
	PD-L1	Inhibiting PD-L1 degradation	Lim et al. (2016)
	EGFR	Inhibiting EGFR degradation	Wan et al. (2019)
	Survivin	Inhibiting survivin degradation	Li et al. (2018)
	HK2	Inhibiting HK2 degradation	Huang et al. (2020)
	FOXM1	Inhibiting FOXM1 degradation	Mao et al. (2019)
	ZEB1	Inhibiting ZEB1 degradation	Zhang et al. (2017)
	p97/VCP	Controling the ubiquitination status of proteins bound to p97/VCP	Cayli et al. (2009)
	ABCA1	Inhibiting ABCA1 degradation	Azuma et al. (2009)
Protein interactions	p53	Promoting p53 nuclear export and degradation	Oh et al. (2006), Zhang et al. (2008)
	CENP-T and CENP-W	Promoting the ubiquitin-dependent degradation of CENP-T and CENP-W	Chun et al. (2013)
	HIF-1α	Stabilizing HIF-1 alpha aerobically by inhibiting HIF-1 alpha prolyl-hydroxylation	Bemis et al. (2004)
	STAMBPL1	Required for the stabilisation and function of STAMBPL1	Chaithongyot and Naumann (2022)
	Malt1 and Carma1	Enhancing the stability of Carma1-Bcl10-Malt1 (CBM) complex	Welteke et al. (2009)
	p27	Promoting cytoplasmic shuttling and subsequent degradation of p27	Tomoda et al. (2002)
	Sec6	Promoting p27 degradation in the cytoplasm via interaction with Jab1	Tanaka and Iino (2014)
	Rig-G	Regulating JAB1 cellular distribution through interacting with this protein and increases the intracellular level of p27	Xiao et al. (2006)
	PGP9.5 (UCH-L1)	Contributing to p27 degradation via its interaction and nuclear translocation with Jab1	Caballero et al. (2002)
	53BP1	Required for mitotic checkpoint activation via its involvement in hyperphosphorylation of 53BP1	Kwak et al. (2005)
	RUNX3	Inducing RUNX3 nuclear export and degradation	Kim et al. (2009)
	Smad7	Inducing Smad7 nuclear export and degradation	Kim et al. (2004)
	TRAF-2	Regulating lysine-63-linked polyubiquitin of TNF receptor-associated-factor 2 which in turn induce TNF-α signaling activation	Wang et al. (2006)
	MDM2	Regulating stabilization of MDM2 through inhibiting MDM2 self-ubiquitination	Zhang et al. (2008)
	HBx	HBx interacts with Jab1 and trigger AP-1 activation	Tanaka et al. (2006)
	LHR precusor	Promoting LHR precusor degradation	Li et al. (2000)
	Smad4	Induces its Smad4 ubiquitylation and degradation	Wan et al. (2002)
	ERα	Increasing ligand-induced ERα degradation	Callige et al. (2005)
	Cyclin E	Promoting Cyclin E degradation	Doronkin et al. (2002)
	CDK2	Inhibiting CDK2 phosphorylation via AKT pathway	Yoshida et al. (2013)
	Myc	Promoting MYC ubiquitination and degradation	Adler et al. (2006)
	MIF	MIF stabilize p27Kip1 by interacting with Jab1; JAB1 inhibits MIF secretion	Kleemann et al. (2000), Lue et al. (2007)
	DNA topoisomerase (topo) II α	Promoting topo II α degradation	Yun et al. (2004)
	ET(A)R and ET(B)R	Promoting ET(A)R and ET(B)R ubiquitination and degradation	Nishimoto et al. (2010)
	Smad5	Inhibiting Smad5-mediated BMP signaling activation	Haag and Aigner (2006)
	PR and SRC-1	Stabilizing PR-SRC-1 complexes	Chauchereau et al. (2000)
	SMYD3	Jab1-SMYD3 complex activates p16INK4a transcription	Mori et al. (2008)
	NCoR	Promoting ubiquitination and proteasome-mediated degradation of NCoR	Lu et al. (2016)
	PAR-2	Promoting PAR-2-induced activation of AP-1	Luo et al. (2006)
	Rad51	Directly affecting Rad51-p53-binding, stabilizing Rad51 and promoting HR DNA repair	Tian et al. (2010)
	CD89	Increasing CD89 surface expression	Bakema et al. (2010)
	CPNE1	JAB1 activates the neuronal differentiation through binding to CPNE1	Yoo et al. (2018)
	TRAF2	JAB1 regulates ubiquitination of TRAF2	Wang et al. (2006)
	Thioredoxin	Stabilizing thioredoxin under oxidative stress	Zhou et al. (2017)
	Id3	Mouse Jab1 was identified to interact with Id3 but the effect remains unclear	Bounpheng et al. (2000)
	NLRP3	Possibly promoting NLRP3 inflammasome activation	Dai et al. (2020)
	Trc8	Trc8 physically interacts with CSN-5 and regulate JAB1 localization	Gemmill et al. (2002)
	PDLIM2	PDLIM2 interacts with CSN5 and regulate CSN activity	Bowe et al. (2014)
	CSNAP	CSNAP binds CSN3, CSN5, and CSN6, thereby regulates the function of CSN complex	Rozen et al. (2015)

More than mediating c-Jun/AP-1 signaling activation, JAB1 also acted as a specific modulator for other transcription factors. For instance, JAB1 interacted with B-cell lymphoma 3(Bcl-3) and facilitated the formation of NF-κB/p50 and a DNA complex. Bcl-3 also activated NF-κB transcriptional activity, as opposed to other members of the inhibitory proteins in IκB family (Dechend et al. 1999). Similarly, JAB1 bound directly to the helix-loop-helix domain of heart and neural crest derivatives expressed 2 (HAND2) and augmented HAND2 transcriptional activity by enhancing HAND2 DNA binding affinity (Dai et al. 2004). Moreover, JAB1 also interacted with Brn-2, a Class III POU transcription factor, and possibly was implicated in regulating neurological functions (Huang et al. 2005). Moreover, binding of JAB1 to HIF-1α resulted in an enhancement of HIF-1α transcriptional activity which could be verified by the increased VEGF expression. However, whether JAB1 potentiates HIF-1α’s DNA-binding activity or just reduces its stability remains obscure (Bemis et al. 2004). In addition, JAB1 interacted with SET and MYND Domain Containing 3 (SMYD3), which functioned as a histone-lysine *N*-methyltransferase. SMYD3 bound to *p16*^*INK4a*^ promoter region containing clustered SMYD3-binding sites and JAB1-SMYD3 complex was shown to activate *p16*^*INK4a*^ transcription (Mori et al. 2008).

### Isopeptidase activity-mediated deNEDDylation and deubiquitination

CSN is similar in structure and architecture to the lid subcomplex of the 26S proteasome which catalyzes degradation of ubiquitin-conjugated proteins in both the cytosol and the nucleus (Wei and Deng 2003; Wolf et al. 2003; Bard et al. 2018). Regulatory particle non-ATPase 11 (Rpn11), a component of lid subcomplex in 26S proteasome, also contains a JAMM domain and is responsible for the proteasome’s cleavage activity (Maytal-Kivity et al. 2002; Verma et al. 2002). JAB1 is the core subunit of the CSN complex responsible for the cleavage of ubiquitin or ubiquitin-like peptides from target proteins (isopeptidase activity). However, JAB1 alone does not have isopeptidase (metalloproteinase) activity indicating other CSN subunits, or perhaps the entire complex, are required for this function (Cope et al. 2002; Cope and Deshaies 2003; Kato and Yoneda-Kato 2009).

A notable role of JAB1 is modulating the activity of cullin-RING ubiquitin ligases (CRLs). CRLs are multi-subunit E3 ubiquitin ligases, which use a cullin (Cul1, Cul2, Cul3, Cul4, Cul5, Cul7, and Cul9) and a RING-box protein (Rbx1 or Rbx2) as the scaffold to connect the E2 enzyme with a specific substrate (Fouad et al. 2019). NEDD8, a ubiquitin-like molecule, is a positive regulator of CRLs. All cullins were shown to be NEDDylated at conserved lysines which were essential for CRL activation and stability (Wu et al. 2005; Schwechheimer 2018; Baek et al. 2020). CSN modulated CRL activity via its deNEDDylation function, thereby regulating the degradation of various CRL-targeted proteins (Shackleford and Claret 2010; Schulze-Niemand and Naumann 2022). For example, NEDDylation of Cul-1 activated SCF^β-TrCP^-mediated ubiquitination of IκBα (Read et al. 2000). JAB1 deNEDDylated Cul-1 and stabilized IkappaB kinase (IκB), thereby significantly attenuating NF-κB activation (Khoury et al. 2007; Majolee et al. 2019). Moreover, downregulation of JAB1 induced proteasome-mediated degradation of the ubiquitin-conjugating enzyme UBC3, which was targeted for ubiquitination and degradation by the cullin-RING ubiquitin ligase SCF^β-TrCP^ (Fernandez-Sanchez et al. 2010). On the other hand, some evidence indicated that CSN could also enhance SCF-CRL activity (Lyapina et al. 2001). For example, JAB1 knockdown inactivated Cul-1 due to enhancement of NEDD8 modification and markedly reduced the basal protein level of the interferon receptor (Muromoto et al. 2013). Moreover, JAB1 was also reported to promote degradation of seven in absentia homolog-1 (SIAH-1) and activate β-catenin pathway via its deNEDDylase activity (Jumpertz et al. 2014). This paradox has been explained in that the deNEDDylation of cullin is necessary to suppress the auto-ubiquitination of F-box proteins and that deNEDDylation is a prerequisite for dynamic cycles of CRL assembly and disassembly, which are also regulated by cullin associated and NEDDylation dissociated 1 (CAND1) (Wee et al. 2005; Cope and Deshaies 2006; Schmidt et al. 2009). CAND1 bound to unNEDDylated cullin-RING box protein complexes and inhibited CRL assembly and activity (Dubiel 2009; Wu et al. 2013). Accumulated evidence showed that CSN exerted a multivalent CRL binding mode and CRLs were differentially sensitive to CSN regulation, both of which increased the complexity of CSN (or JAB1) in regulating CRL activity (Schulze-Niemand and Naumann 2022).

The CSN complex also possesses de-ubiquitination activity. For instance, JAB1 was reported to modulate ubiquitin-dependent protein sorting into exosomes by mediating de-ubiquitination of HSP70 and Snail (Liu et al. 2009; Wu et al. 2009). JAB1 could also de-ubiquitinate and stabilize PD-L1 which, in turn, leaded to T cell suppression (Lim et al. 2016). Moreover, JAB1 directly interacted with angiopoietin-like protein 2 (ANGPTL2) and attenuated its ubiquitin-mediated degradation through de-ubiquitylation (Xie et al. 2021).

### Other functions mediated by protein interactions

In addition to transcriptional co-activation and isopeptidase activity, JAB1 possesses a broad series of functions mediated by protein interactions (Table 1). As mentioned above, JAB1 controls cullin-dependent protein degradation through regulating cullin deNEDDylation. However, accumulated studies have revealed more mechanisms on JAB1-involved regulation of protein degradation, which are possibly independent to its isopeptidase activity. First, JAB1 regulated protein degradation by controlling protein subcellular translocation. JAB1 bound to p27 and promoted p27 shuttling from the nucleus in a Exportin 1 (XPO1)-dependent manner, which in turn accelerated p27 degradation through the ubiquitin-dependent proteasome pathway (Tomoda et al. 1999, 2002). Similarly, JAB1 also induced nuclear export and degradation of p53 (Oh et al. 2006; Zhang et al. 2008), RUNX3 (Kim et al. 2009), and Smad7 (Kim et al. 2004). Another notable role of JAB1 is to affect protein mortification. For instance, JAB1 regulated lysine-63-linked polyubiquitin of TNF receptor-associated-factor 2 which in turn induced TNF-α signaling activation (Wang et al. 2006). JAB1 also regulated stabilization of mouse double minute 2 homolog (MDM2) through inhibiting MDM2 self-ubiquitination (Zhang et al. 2008). Moreover, JAB1 also regulates protein transmembrane transport. For example, JAB1 controlled autocrine MIF-mediated Akt signaling by inhibiting MIF secretion (Lue et al. 2007). Furthermore, JAB1 can also function as a mediator in stabilizing or competing with protein interactions. For example, JAB1 interacted with both the progesterone receptor (PR) and the steroid receptor coactivator 1 (SRC-1) and stabilized the PR-SRC-1 complex (Chauchereau et al. 2000). In contrast, JAB1 competed with p53 to bind directly to the oxygen-dependent death domain of HIF-1α, resulting in stabilization of HIF-1α by blocking hypoxia dependent degradation (Bae et al. 2002).

## JAB1 in neurodevelopment

**Fig. 3:**
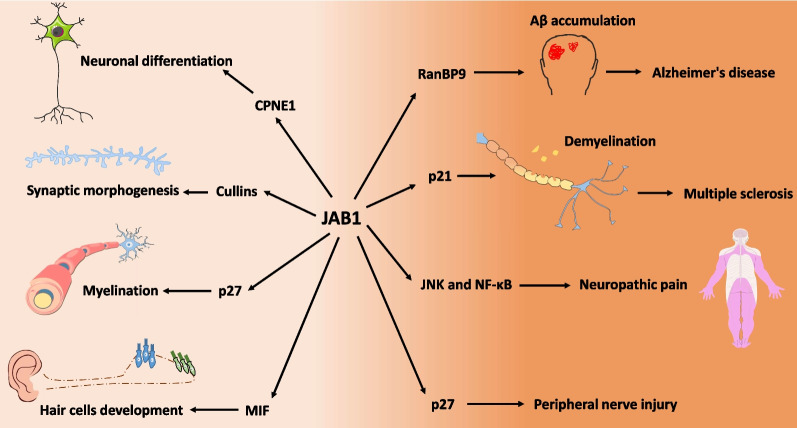
Schematic overview of JAB1 in neurodevelopment and neurological diseases. The downstream factors and potential mechanisms of JAB1 in different processes are depicted

Increasing evidence supports that JAB1 is functional in neurodevelopment. In this section, we will review the roles and underlying mechanisms of JAB1 in the processes of neuronal differentiation, synaptic morphogenesis, myelination, and hair cells development (Fig. 3), and also discuss potential implications of JAB1 in neurodevelopmental disorders.

### Neuronal differentiation

Copines (CPENs) are a family of membrane-anchored proteins that are highly evolutionarily conserved in sequence and structure within eukaryotes (Creutz et al. 1998; Tomsig et al. 2003). CPENs function in a myriad of cellular processes and signaling pathways, such as membrane transport, lipid second messenger regulation, GTPase activation and protein phosphorylation (Mukhopadhyay et al. 2017; Tang et al. 2021). An increasing number of studies have demonstrated that CPNEs participate in neuronal differentiation. CPNE1 is abundantly expressed in neural stem cells (NSCs) and immature neurons in human and mouse brains and CPNE1 deficiency could decrease the proliferation and multi-lineage differentiation potential of NSCs by downregulating the mTOR signaling pathway (Kim et al. 2018). In a hippocampal progenitor cell line HiB5, CPNE1 was increased at the early stage of neuronal differentiation while CPNE1 knocked-down leaded to a defect in PDGF-mediated neurite outgrowth (Park et al. 2012). Park et al. demonstrated that the C2 domains of CPEN1 mediated neuronal differentiation by regulating Akt phosphorylation (Park et al. 2014). AKT has been shown to be a crucial cassette for signal transduction during neuronal development (Read and Gorman 2009; Zhong 2016). Moreover, 14-3-3γ, a phospho-binding protein, interacted with CPNE1 and acted as a coordinator of CPNE1 in regulating HiB5 differentiation (Cheal Yoo et al. 2017). Similar to 14-3-3γ, JAB1 specifically bound to the CA2 domain of CPEN1. Overexpression of JAB1 enhanced CPNE1-dependent differentiation of HiB5 indicating a synergistic effect of JAB1 and CPNE1 during neuronal differentiation even though the underlying mechanism deserves further clarification (Yoo et al. 2018).

JAB1 is also critical for photoreceptor neuron differentiation. Rod and cone photoreceptors are specialized neurons found in the retina that function in the initial step of vision through converting light into electrical signals to the brain for processing (Molday and Moritz 2015). In *Drosophila*, JAB1 is highly expressed in rod cells and accumulates in the developing optic lobe neuropil. JAB1 was shown to affect rod and cone cell development by regulating lamina glial cell migration into the target region in a COP9 signalosome dependent pathway (Suh et al. 2002). At present, it still remains unclear whether JAB1 functions in connections of optic ganglia by regulating photoreceptor development in other species except for *Drosophila*. However, in mouse neural stem cells (NSCs), Wang et al. identified JAB1 as a potential modulator downstream of the melanopsin/transient receptor potential channel 6 (TRPC6) pathway which directed light-induced NSC differentiation (Wang et al. 2019a). Moreover, JAB1 also interacted with Brn-2 and possibly activated Brn-2 downstream signaling pathway in neuronal differentiation (Huang et al. 2005). Brn-2 is a POU domain transcription factor which is crucial for the differentiation of fibroblasts to functional excitatory cortical neurons (Miskinyte et al. 2017).

### Synaptic morphogenesis

Dendritic spine morphogenesis is a fundamental process in synapse formation and maturation, which is crucial for synaptic plasticity and function. Dendritic abnormalities are featured pathology in various neurological disorders (Kaufmann and Moser 2000; Dierssen and Ramakers 2006; Knobloch and Mansuy 2008). In *Drosophila*, a JAB1 homozygous mutant could lead to aberrant dendritic morphology in dendritic arborization (DA) sensory neurons exhibiting shorter and less dendritic branching. Moreover, normally highly branched ddaC neurons also developed significantly fewer branches and a shrunken dendritic tree due to JAB1 deficiency. Mechanistically, JAB1 functions in synaptic morphogenesis possibly through regulating cullin NEDDylation and cullin-mediated proteins degradation in a CSN-dependent pathway (Djagaeva and Doronkin 2009).

### Myelination

Myelination is developed as an ingenious strategy to segregate neuronal axons from environmental insult and to promote conduction of electric action potentials down the axons. In peripheral nerves, Schwann cells (SCs) produce lipid-rich layers of myelin to wrap around the neuronal axons. Axonal sorting is a crucial event in myelination which requires SCs proliferation, differentiation, and contact with axons (Williamson and Lyons 2018). In this process, SCs proliferate and expand cellular extensions into bundles of unsorted axons and establish the one-to-one relationship with individual axons (Webster et al. 1973; Jessen et al. 2015; Min et al. 2021). Mice with conditional knock-out of *JAB1* in SCs manifested impaired axonal sorting and motor dysfunction. Axonal sorting is supported by proper SC differentiation (Jessen and Mirsky 2005) while JAB1 deletion led to delayed or arrested SC differentiation which was associated with abnormally increased level of p27 (Porrello et al. 2014). Increased levels of p27 have been reported to cause cell cycle arrest in oligodendrocytes and SCs (Casaccia-Bonnefil et al. 1999; Li et al. 2011). However, genetic depletion of p27 restored SC number and axonal sorting in JAB1 deficiency SCs. This evidence indicates that JAB1 regulates SC proliferation and axonal sorting through the p27-associated signaling pathway (Porrello et al. 2014).

### Hair cells development

Hair cells (HCs) function as the specialized sensory receptors for both the auditory and vestibular systems in the ears of animals. Inner ear hair cells can transduce sound-evoked mechanical vibrations into electrical signals which are then relayed to the brain (Wang et al. 2015). HC development and innervation by the vertebrate statoacoustic ganglion (SAG) are crucial for the auditory function and involve a plethora of signaling pathways (Wang et al. 2019b). Macrophage migration inhibitory factor (MIF) acts as neurotrophic cytokines during the earliest stages of inner ear development. JAB1 has been reported to control autocrine MIF-mediated Akt signaling by inhibiting MIF secretion (Mcginley et al. 2021). More than that, JAB1 is also a downstream effector of MIF during inner ear hair cell development in zebrafish (Wang et al. 2016b). However, the potential function of JAB1 in hair cell development in mammals needs further verification.

### Putative roles of JAB1 in neurodevelopmental disorders

Considering the vital functions of JAB1 and its effectors in different neurodevelopmental processes, dysregulation or dysfunction of JAB1 may contribute to some neurodevelopmental disorders. First, this could be partially supported by a clinical case in which a patient with a 1.4 Mb interstitial deletion at the 8q13.1-q13.2 locus (JAB1 contained) exhibited inferior cerebellar vermian hypoplasia and digital anomalies (Mordaunt et al. 2015). Moreover, JAB1 may be involved in the pathogenesis of autism through affecting c-Jun activation. Aberrant increase of c-Jun was reported in an autism-like mouse model (Tripathi et al. 2009), and c-Jun activation could also induce a disordered inflammatory response in the central nervous system (Shimoyama et al. 2019) which was featured in the autism brain (Bjorklund et al. 2020; Roe 2022). Furthermore, as a component of the CSN complex, JAB1 potentially participated in the pathogenesis of Down syndrome (Peyrl et al. 2002) and Smith–Magenis syndrome (Elsea et al. 1999).

## JAB1 in neurological diseases

### Alzheimer’s disease

As the dominating contributor to dementia, Alzheimer's disease (AD) is the most prevalent neurodegenerative disorder. β-amyloid (Aβ) and hyperphosphorylated Tau are two of the most featured pathological proteins that lead to senile plaques and neurofibrillary tangles, respectively in AD brains. Aβ peptides are produced from amyloid precursor protein (APP) through sequential cleavages by β-secretase and γ-secretase. Ran-binding protein (RanBP) is a scaffolding protein implicated in a variety of signal transduction pathways (Suresh et al. 2012). RanBP9 interacted with APP and BACE1, thereby enhancing β-secretase processing of APP by accelerating APP internalization and interaction with BACE1 (Lakshmana et al. 2009). Transgenic mice overexpressing RanBP9 exhibited increased Aβ plaque burden in the brain (Lakshmana et al. 2012) while knockdown of endogenous RanBP9 significantly reduced Aβ production in Chinese hamster primary neurons (Lakshmana et al. 2009). Similar to RanBP9, JAB1 could also increase the Aβ level by promoting β-secretase processing of APP while down-regulation of JAB1 reduced Aβ generation, indicating the vital role of JAB1 in regulating Aβ production (Wang et al. 2013). JAB1 was shown to be increased in the brains of AD patients and APP/PS1 transgenic mice (AD mouse model); JAB1 overexpression strongly increased the RanBP9 protein level by increasing its half-life (Wang et al. 2013); however, whether JAB1 regulates RanBP9 subcellular translocation or its degradation-associated modifications is unknown. Consistently, JAB1 overexpression in APP/PS1 transgenic mice significantly increased amyloidogenic processing of APP, and reduced spinophilin (the marker of dendritic spines) in both the cortex and the hippocampus, leading to significant defects in learning and memory skills (Wang et al. 2015). Taken together, this evidence implied that JAB1 could aggravate Aβ pathology and cognitive decline by increasing RanBP9 stability in AD brain (Fig. 3).

Furthermore, JAB1 may be implicated in AD progression through other pathways. For instance, JAB1 participated in unfolded protein responses by interactions with ER-resident transmembrane kinase-endoribonuclease inositol-requiring enzyme 1 (IRE1) in response to ER stress (Oono et al. 2004), a well-known abnormal phenomena in the context of AD (Uddin et al. 2021). IRE1 is an ER-located kinase and endoribonuclease that functions as a major transducer under ER stress. In human brains, IRE1 activation was reported to exacerbate progression of AD histopathology (Duran-Aniotz et al. 2017). JAB1 also interacted with ubiquitin C-terminal hydrolase L1 (UCH-L1, also termed as PGP9.5) (Caballero et al. 2002), which was found to be highly expressed in the cerebral cortex and hypothalamus (Sharma et al. 2020). UCHL1 affected Aβ production by promoting APP ubiquitination and lysosomal degradation (Zhang et al. 2014). However, whether JAB1 regulates APP modification and processing via UCHL1 remains unclear so far.

### Multiple sclerosis

Multiple sclerosis (MS) is an autoimmune-mediated neurodegenerative disease with the main and distinguishing feature of inflammatory demyelination with axonal transection (McGinley et al. 2021). In MS, demyelination caused by focal lymphocytic infiltration into the central nervous system (CNS) can lead to permanent damage or deterioration of the nerves in CNS (Hauser and Cree 2020). Arising as the most common cause of non-traumatic neurologic disability in young adults, MS affects more than 2.5 million people worldwide (McGinley et al. 2021; Rodriguez Murua et al. 2022). In MS patients, JAB1 was shown to be reduced in oligodendrocytes (Rivellini et al. 2022). Moreover, oligodendrocyte-conditional JAB1 mutant mice exhibited MS-like pathologies, such as demyelination, fostered chronic inflammation, and oxidative stress in the CNS. Oligodendrocyte lacking JAB1 expression developed a premature senescence phenotype with deteriorative DNA damage and defective DNA repair while deletion of p21 could ameliorate these JAB1 deficiency-induced phenotypes (Rivellini et al. 2022). This evidence indicates that JAB1 deficiency-induced cellular senescence may be a crucial cause to MS (Fig. 3). JAB1 was reported to regulate cellular senescence by affecting cyclin dependent kinase 2 (CDK2) translocation. Deletion of JAB1 in mouse embryonic fibroblasts suppressed cell proliferation, and induced premature senescence characterized by enhancing senescence-associated-β-galactosidase activity and increased expression of CDK inhibitors (Tsujimoto et al. 2012). JAB1 interacted with CDK2 and inhibited CDK2 phosphorylation. Deletion of JAB1 increased the phosphorylation of CDK2 by Akt, resulting in accumulated CDK2 together with cyclin E in cytoplasm (Yoshida et al. 2013). However, whether the JAB1-CDK2 signaling axis is implicated in the pathogenesis of MS needs further exploration.

### Neuropathic pain

Neuropathic pain, a chronic pain condition, is commonly caused by a lesion or dysfunction in the somatosensory nervous system (Baron et al. 2010). Neuropathic pain is considered to be the consequence of aberrant excitability of dorsal horn neurons evoked by peripheral sensory inputs, which is clinically featured as hyperalgesia and allodynia (Finnerup et al. 2021); however, the mechanism has not been fully elucidated. Interestingly, in a neuropathic pain rat model induced by chronic constriction injury (CCI), JAB1 was mostly increased in the neurons in the dorsal root ganglion and spinal cord (Chen et al. 2016). Moreover, phosphorylation of JNK1 and p65 (NF-κb) were also upregulated in this model. Importantly, down-regulation of JAB1 could significantly reduce phosphorylated JNK1 and p65, and effectively ameliorate neuropathic pain-associated behavior shown by the increased values of the paw withdrawal latency and the paw withdrawal threshold (Chen et al. 2016). These results implied that JAB1 was implicated in the pathogenesis of neuropathic pain via the JNK and NF-κB pathway (Fig. 3), but whether JAB1 affects the phosphorylation of JNK and p65 or regulates the degradations of phosphorylated JNK1 and p65 remains unclear.

### Peripheral nerve injury

Peripheral nerve injury caused by traumatic damage or complications of other diseases is increasing as a devastating clinical and public health problem that often gives rise to significant functional morbidity and permanent disability (Alvites et al. 2018). Patients with peripheral nerve injury can suffer severe and persistent pain, or even total loss of sensation in the part of the body influenced by the damaged nerve (Burnett and Zager 2004). In a rat model with sciatic nerve injury, JAB1 was shown to be increased from 12 h to 7 days post-injury (Cheng et al. 2013). Consistently, p27 also presented a significant change contrary to that of JAB1 as JAB1 was reported to regulate p27 subcellular translocation and degradation (Tomoda et al. 2002). Moreover, the interaction between JAB1 and p27 was also identified in the sciatic nerve (Cheng et al. 2013). These results illustrated that JAB1 and p27kip1 may be involved in the pathology of the sciatic nerve after injury (Fig. 3) but how JAB1 acts in this model remains unclear. Moreover, SCs within the peripheral nervous system possess a remarkable regeneration capacity which is crucial for nerve regeneration and functional recovery following traumatic injuries (Nocera and Jacob 2020; Min et al. 2021). Considering the pivotal roles of JAB1 in SC proliferation and axonal sorting, which have been described above, JAB1 may function as a potential target for nerve repair by promoting remyelination and axonal growth.

## Challenges and prospects on drug development by targeting JAB1

Considering the participations of JAB1 in diverse pathological processes, JAB1 can be developed as a biomarker or therapeutic target in various neurological disorders. However, so far there is no specific JAB1-targeting drug has been developed in clinical trials, as inconsistent expression change and bidirectional roles of JAB1 in different diseases should be taken into consideration. For example, JAB1 was increased in AD brains (Wang et al. 2013) but was reduced in oligodendrocytes of MS patients (Rivellini et al. 2022). In AD model, reduced JAB1 is beneficial for inhibiting Aβ production while JAB1 deficiency can also cause MS-like pathologies. Hence, simply changing JAB1 levels may not be an effective strategy as this could produce unpredictable side effects. For instance, as an oncogene, it remains unclear whether exogenous upregulation of JAB1 may increase the risk of tumor development. Nevertheless, for some acute disorders, interventions for JAB1 levels in some specific local tissues may be an alternative method to ameliorate the deteriorative symptoms. For example, down-regulation of JAB1 in dorsal root ganglion can effectively improve hyperalgesia in a CCI-induced rat model (Chen et al. 2016). However, another concern on intervening in JAB1 expression is that there are limited studies investigating the regulatory pathways involved in JAB1 expression in the nervous system. As we have summarized above, the expression of JAB1 is regulated at multiple levels. However, it has been rarely studied as to whether these mechanisms are functional in neurodevelopment or neurological diseases. Hence, it still remains a challenging to modulate JAB1 expression and requires further studies in the future.

Some chemicals targeting the interactions of JAB1 with its downstream effectors may bring more prospects for clinical application. JAB1 functions by interacting with different proteins in various biological pathways, hence, modifying a single specific activity of JAB1 or regulating the interaction between JAB1 and one protein through peptides or small molecular compounds may provide a more precise therapeutic strategy. For instance, Azaindoles, a Zinc-binding small-molecule, was reported to inhibit JAB1 deNEDDylation activity by interacting with the active-site zinc ion of JAB1 (Altmann et al. 2017). Similarly, thiolutin can also inhibit JAB1 metalloprotease activity (Lauinger et al. 2017) and has shown potential benefits for treatment of NLRP3-associated inflammatory diseases (Ren et al. 2021). Besides, caffeic acid phenethyl ester suppressed the interaction between NLRP3 and CSN5 and inhibited NLRP3 inflammasome activation (Dai et al. 2020). CSN5i-3, a potent, selective and orally available inhibitor of JAB1 exhibited anti-tumour activity by traping CRLs in the neddylated state, which leaded to CRLs inactivation (Schlierf et al. 2016; Xiao et al. 2019). On the other hand, although accumulating evidence has supported JAB1-targeted chemicals provide more potential for medical application, detailed pharmacokinetics and safety evaluation of such compounds should also be addressed in future studies.

## Conclusion

JAB1 has been identified as a vital regulator involved in various signaling pathways. More importantly, mounting evidence supports that JAB1 plays crucial roles in neuronal differentiation, synaptic morphogenesis, myelination, and hair cell development, and is also implicated in the pathogenesis of some neurological diseases. JAB1 downregulation exerts potential benefits for AD and neuropathic pain treatment, but may also increase the risk for MS development. Interventions for JAB1 expression levels have shown therapeutic potential for some neurological diseases, but specific molecules interfering with the interaction of JAB1 with target proteins may have a brighter future.

## Data Availability

Not applicable.

## Abbreviations

c-Jun: Jun proto-oncogene
Bcl-3: B cell leukemia/lymphoma 3
HAND2: Heart and neural crest derivatives expressed 2
LFA-1: Lymphocyte function-associated antigen 1
Brn-2 (Pou3f2): POU class 3 homeobox 2
5-HT(6)R: 5-Hydroxytryptamine(6) receptor
Fank1: Fibronectin type III and ankyrin repeat domains 1
MIF: Macrophage migration inhibitory factor
E2F1: E2F transcription factor 1
MSRA: Methionine sulfoxide reductase A
ANGPTL2: Angiopoietin like 2
PD-L1: Programmed death-ligand 1
EGFR: Epidermal growth factor receptor
HK2: Hexokinase 2
FOXM1: Forkhead box M1
ZEB1: Zinc finger E-box binding homeobox 1
p97/VCP: Valosin containing protein
ABCA1: ATP binding cassette subfamily A member 1
CENP-T: Centromere protein T
CENP-W: Centromere protein W
HIF-1α: Hypoxia-inducible factor 1 α
STAMBPL1: STAM binding protein like 1
Malt1: MALT1 paracaspase
Carma1: CARD-containing MAGUK protein 1
Sec6(EXOC3): Exocyst complex component 3
Rig-G(IFIT3): Interferon induced protein with tetratricopeptide repeats 3
PGP9.5 (UCH-L1): Ubiquitin C-terminal hydrolase L1
53BP1: P53 binding protein 1
RUNX3: RUNX family transcription factor 3
SMAD(4,5,7): SMAD family member (4,5,7)
TRAF2: TNF receptor associated factor 2
MDM2: Mouse double minute 2 homolog
HBx: Hepatitis B virus X
LHR: Lethal hybrid rescue
Erα: Estrogen receptor 1 α
CDK2: Cyclin dependent kinase 2
ET(A/B)R: Endothelin type A and B
PR: Progesterone receptor
SRC-1: Steroid receptor coactivator-1
SMYD3: SET and MYND domain containing 3
NCOR: Nuclear receptor corepressor
PAR-2: Protease-activated receptor-2
RAD51: RAD51 recombinase
CPNE1: Copine 1
TRAF2: TNF receptor associated factor 2
ID3: Inhibitor of DNA binding 3
NLRP3: NLR family pyrin domain containing 3
TRC8/RNF139: Ring finger protein 139
PDLIM2: PDZ and LIM domain 2
CSNAP: CSN acidic protein
